# Urethra Shake or You Don’t: Characterizing the Post-Void Shudder

**DOI:** 10.5334/tohm.1307

**Published:** 2026-11-09

**Authors:** Mered Parnes

**Affiliations:** 1 Pediatric Movement Disorders Clinic, Division of Pediatric Neurology and Developmental Neuroscience, Baylor College of Medicine and Texas Children’s Hospital, Houston TX, USA

**Keywords:** shiver, chill, shudder, micturition, urination, reflex

## Abstract

**Background:**

On occasion, upon emptying the bladder, some individuals experience a brief, patterned, paroxysmal tremor event predominantly involving the axial upper body. This phenomenon has never been described in the medical literature. This report serves to define and characterize the phenomenology, prevalence and distribution of these events.

**Methods:**

A 12-question survey was distributed to 2,083 pediatric physicians at Baylor College of Medicine. Comparisons between groups were performed using p-value < 0.05.

**Results:**

There were 413 respondents; median age was 41 years, and 309 (74.8%) were female. A total of 124 respondents (30%) experience post-void shudder, consisting of 68/309 females (22%) and 56/104 males (53.8%) (p < 0.001). Three hundred and eighteen out of 413 (77%) experience similar shudders sporadically and without a known trigger.

**Discussion:**

Shudders are a distinct type of involuntary physiologic reflex event. Post-void shudder is common, occurring in up to 30% of the population and affecting both men and women. Sporadic physiologic shudders, shuddering attacks, and post-void shudder share a common phenomenology.

**Highlights:**

Post-void shudder (PVS) is a brief, patterned, paroxysmal tremor event involving the axial upper body, that occurs upon emptying the bladder. This report is the first to describe this common phenomenon. Survey results (413 respondents) indicate that PVS occurs in up to 30% of adults, men more often than women.

## Introduction

The aim of this report is to describe a common and previously uncharacterized condition, which I define here as *post-void shudder* (PVS).

This phenomenon is no doubt familiar to many who are reading this article. It consists of a *shudder*, defined here as a brief, patterned, paroxysmal tremor event predominantly involving the axial upper body, that in this case occurs immediately upon emptying of the bladder. The event lasts about one second and may take the form of 2–5 rotatory oscillations about the vertical axis of the neck and torso. There may be associated quick elevations of the shoulders or isometric contraction of the upper body. Very similar events have been described in the setting of shuddering attacks in children [1].

Although it has never been characterized in the medical literature, PVS was described by stand-up comedian George Carlin on his 1975 album *An Evening With Wally Londo*, and again in 1994 in the syndicated question-and-answer newspaper column The Straight Dope after a question was submitted on the subject [2,3]. In the author’s lighthearted response, the medicalese term “post-micturition convulsion syndrome” was coined, no doubt for comedic effect. I suggest that another term would be more suitable, as these events neither qualify as a syndrome nor are best described as a convulsion.

This pilot study was designed to determine the prevalence and distribution of these events.

## Methods

Using a protocol approved by the Baylor College of Medicine Institutional Review Board, a 12-question survey (Figure 1) was created in Research Electronic Data Capture (REDCap) and distributed via listserv electronic mailing list to 2,083 faculty, fellows and residents in the Department of Pediatrics at Baylor College of Medicine. Responses were elective and were collected anonymously.

**Figure 1:**
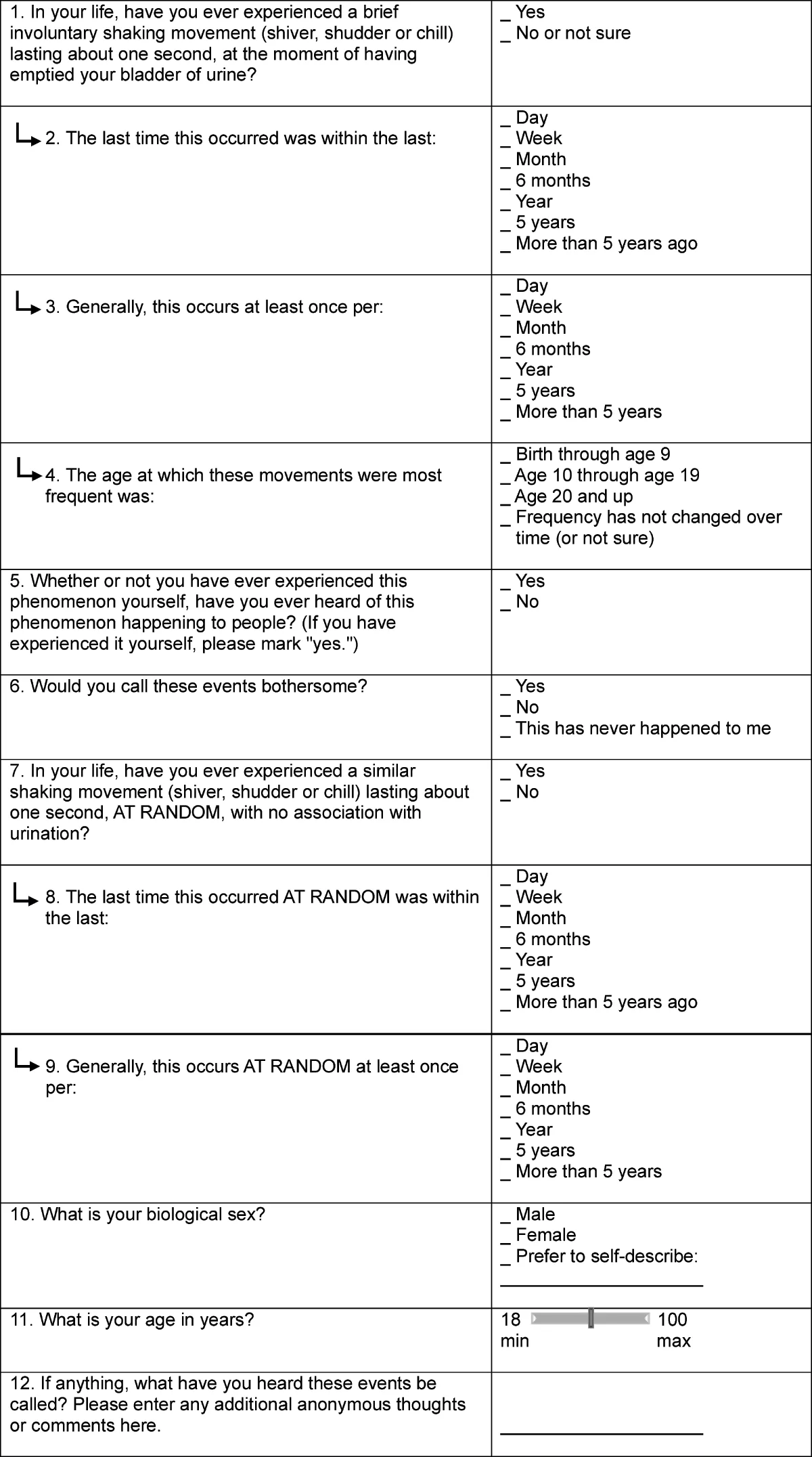
Post-void shudder questionnaire.

Participant characteristics and survey responses were summarized using frequencies and proportions for categorical variables and median with interquartile range (IQR) for continuous variables. Comparisons between groups were performed using Pearson’s chi-square test or Fisher’s exact test for categorical variables, and the Wilcoxon rank-sumtest for continuous variables, as appropriate. Analyses comparing PVS-related characteristics were restricted to participants reporting post-void shuddering, and analyses of sporadic shudder characteristics were restricted to participants reporting sporadic shudders. Because the analyses were exploratory, no adjustments were made for multiple comparisons. Statistical significance was defined as a two-sided p-value <0.05. Analyses were conducted using Stata version 19.5 (StataCorp LLC, College Station, TX).

## Results

Descriptive statistics for the participants are presented in Table 1. Responses from 413 individuals were received and included in the analysis. Median age was 41 years (IQR 33-50). Three hundred and nine respondents (74.8%) were female. One hundred twenty-four respondents (30%) experience PVS. Sixty-eight out of 309 female respondents (22%) and 56/104 male respondents (58.8%) experience PVS (p < 0.001). Three hundred and eighteen out of 413 (77%) experience similar shudders sporadically and without a known trigger. After adjustment for age and sex, there was not a significant association between PVS and sporadic shudders (adjusted OR 0.66, 95% CI 0.38, 1.17; p = 0.16).

**Table 1:** Participant characteristics and survey results.


	TOTAL

	N = 413

Age (years), median (IQR)	41.0 (33.0, 50.0)

Sex	

Female	309/413 (74.8)

Male	104/413 (25.2)

Post-void shudder	

No	289/413 (70.0)

Yes	124/413 (30.0)

Most recent PVS†	

Day	6/122 (4.9)

Week	23/122 (18.9)

Month	26/122 (21.3)

6 months	35/122 (28.7)

Year	20/122 (16.4)

5 years	7/122 (5.7)

>5 years	5/122 (4.1)

PVS frequency†	

Day	5/121 (4.1)

Week	24/121 (19.8)

Month	30/121 (24.8)

6 months	36/121 (29.8)

Year	19/121 (15.7)

5 years	3/121 (2.5)

>5 years	4/121 (3.3)

Peak PVS age†	

Age 0–9	5/121 (4.1)

Age 10–19	13/121 (10.7)

Age ≥20	19/121 (15.7)

No change/not sure	84/121 (69.4)

Ever heard of phenomenon	

No	262/413 (63.4)

Yes	151/413 (36.6)

Would you call these events bothersome?	

No	123/124 (99.2)

Yes	1/124 (0.8)

Sporadic shudder	

No	95/413 (23.0)

Yes	318/413 (77.0)

Most recent sporadic shudder	

Day	14/314 (4.5)

Week	43/314 (13.7)

Month	97/314 (30.9)

6 months	79/314 (25.2)

Year	47/314 (15.0)

5 years	22/314 (7.0)

>5 years	12/314 (3.8)

Sporadic shudder frequency	

Day	6/314 (1.9)

Week	35/314 (11.1)

Month	93/314 (29.6)

6 months	95/314 (30.3)

Year	48/314 (15.3)

5 years	21/314 (6.7)

>5 years	16/314 (5.1)


Values are presented as n/N for categorical variable and as median (IQR) for continuous variables. Parentheses for categorical variables refer to percentages. † Among participants reporting post-void shuddering. IQR interquartile range; PVS, post-void shudder.

For most individuals surveyed, PVS occurs once weekly to once annually, although 5 respondents (4.1%) report experiencing at least one per day. Sporadic shudders occur with similar frequency. Most participants did not notice a change in the frequency of PVS over time. Six point six percent of those surveyed who have not experienced PVS had heard of the phenomenon. Only one participant with PVS described it as bothersome.

There was a modest but significant difference (p = 0.01) in the age of the respondents when stratified by sex, with median age of female respondents of 40 (IQR 32-49) and of males of 44.5 (IQR 36-52.5).

Twenty-one participants commented in Question 12 that they know PVS as, or have heard it called, “pee shivers” or a variation thereof (pee-pee shivers, pee shakes, post-pee shivers, or a slightly less genteel variant). Four participants answered that PVS is more likely to occur when they have delayed urinating and the bladder is very full.

## Discussion

The findings of the survey suggest that PVS is quite common, and affects about 2.5 times as many men as it does women.

I propose that “pee shivers,” as the survey confirms these events colloquially are called, belong to a class of involuntary physiologic reflex events in humans that appear purposeless, may serve some vestigial evolutionary objective, and can have a predictable situational association, like contagious yawning, pre-performance emesis, emotional piloerection (goosebumps), and emotional facial blushing [4,5,6,7].

The means by which this phenomenon is mediated is not yet known. Regulation of urine storage and voiding is accomplished by sophisticated hierarchical systems which involve spinal cord, peripheral autonomic ganglia, brainstem, and brain circuitry. In the lower urinary tract, urothelial mechanoreceptors detect the degree of bladder distention and transmit this information via pelvic nerve afferents originating in the lumbosacral dorsal root ganglion. This information is transmitted primarily by myelinated Aδ fibers to the midbrain periaqueductal gray (PAG), where it is relayed to the pontine micturition center, insular cortex, and other areas involved in reflex and volitional control of micturition [8,9,10].

I propose that post-void shudders are triggered by visceral sensory information received from the bladder or urethra as voiding ends, and that the patterned motor output is mediated reflexively by brainstem and autonomic pathways. Additional studies are needed to elucidate the underlying neuroanatomic and neurophysiologic basis of shuddering. With regard to the difference in frequency between men and women, apart from differences in anatomy, a potential explanation could be related to the standing versus seated position.

It is common for similar shudders to occur spontaneously and seemingly at random. This also has not yet been reported in the literature, outside of the shuddering attacks described in the seminal article by Venasse et al in 1976, and in more recent reports [1,11,12,13]. These sporadic physiologic shudders, PVS, and childhood shuddering attacks appear to differ only in frequency and duration, as the phenomenology of the events themselves is identical. The shudders in shuddering attacks can occur up to 100 times daily and can sometimes last over 5 seconds [1,12,13]. Instructive videos of a young child with shuddering attacks are included in the paper by Tibussek and colleagues [13]. Of course, shuddering attacks and spontaneous physiologic shudders have no known association with micturition. Anecdotally, post-void shudder may also occur in infants and children, although this has not yet been studied.

The language surrounding shuddering in English vernacular is imprecise and is a potential impediment to its study. In the proper context, the nouns *shiver* and *chill* can be used as synonyms for shudder. However, shudder is preferred here due to its specificity. Consider that while the verb “to shiver” may at times be used to mean “to shudder,” it also can describe an entirely different phenomenon, the more persistent generalized tremor in the context of a mounted thermoregulatory response to being cold or febrile [14,15]. This is characterized by an increase in tonic muscle contraction and superimposed waxing and waning 8–10 Hz tremor that begins in muscles of the trunk and spreads distally to involve the limbs, and may include prominent tremor of the jaw (“chattering” of the teeth) [16,17,18,19,20,21]. Shuddering and thermogenic shivering are phenomenologically distinct.

Similarly, while the noun “chill” may refer to a shudder, the same word also describes a patterned brief sensory phenomenon consisting of a cold feeling traveling down the back (a “cold chill”), and the plural “chills” describes a third event type, an episode of feeling cold for periods of minutes in the context of fever, which together with thermogenic shivering is called *rigors*, or “shaking chills” [22].

I acknowledge that the generalizability of the findings reported here might be affected by several factors, including potential sampling bias due to the sample size, sex distribution, and population surveyed, response bias due to the use of an elective questionnaire, and recall bias due to the retrospective nature of the survey. The proportion of female respondents mirrors the national distribution of pediatricians in the United States [23]. Future studies would benefit from a prospective design, a more diverse and balanced population, inclusion of pertinent medical and family history, and ideally objective measures of duration, frequency and somatic distribution of the events.

This pilot study was designed to initiate a discussion regarding this common reflex action. I invite readers to comment and provide input to further this effort.

## References

[1] Vanasse M, Bedard P, Andermann F. Shuddering attacks in children: an early manifestation of essential tremor. Neurology. 1976;26(11):1027–1030. DOI: 10.1212/WNL.26.11.1027 988508

[2] Carlin G. An Evening with Wally Londo Featuring Bill Slaszo. Little David/Atlantic; 1975.

[3] Adams C. What causes “piss shiver”? The Straight Dope. July 22, 1994. Accessed September 18, 2026. https://www.straightdope.com/21342202/what-causes-piss-shiver

[4] Cordoni G, Favilli E, Palagi E. Earlier than previously thought: Yawn contagion in preschool children. Dev Psychobiol. 2021;63(5):931–944. DOI: 10.1002/dev.22094 33506489

[5] Cleghorn RA, Brown WT, Melville PH. Psychogenesis of Emesis. Can Psychiatr Assoc J. 1964;9(4):299–311. DOI: 10.1177/070674376400900405 14178679

[6] Katahira K, Kawakami A, Tomita A, Nagata N. Volitional Control of Piloerection: Objective Evidence and Its Potential Utility in Neuroscience Research. Front Neurosci. 2020;14:590. DOI: 10.3389/fnins.2020.00590 PMC7290233 32581701

[7] Drummond PD, Lance JW. Facial flushing and sweating mediated by the sympathetic nervous system. Brain J Neurol. 1987;110(Pt 3):793–803. DOI: 10.1093/brain/110.3.793 3580835

[8] Griffiths D. Neural control of micturition in humans: a working model. Nat Rev Urol. 2015;12(12):695–705. DOI: 10.1038/nrurol.2015.266 26620610

[9] De Groat WC, Griffiths D, Yoshimura N. Neural Control of the Lower Urinary Tract. Compr Physiol. 2015;5(1):327–396. DOI: 10.1002/j.2040-4603.2015.tb00596.x PMC4480926 25589273

[10] Benarroch EE. Neural control of the bladder: recent advances and neurologic implications. Neurology. 2010;75(20):1839–1846. DOI: 10.1212/WNL.0b013e3181fdabba 21079186

[11] Holmes GL. Shuddering Attacks: Evaluation Using Electroencephalographic Frequency Modulation Radiotelemetry and Videotape Monitoring. Am J Dis Child. 1986;140(1):72. DOI: 10.1001/archpedi.1986.02140150074041 3942112

[12] Kanazawa O. Shuddering Attacks--Report of Four Children. Pediatr Neurol. 2000;23:421–424. DOI: 10.1016/S0887-8994(00)00205-8 11118798

[13] Tibussek D, Karenfort M, Mayatepek E, Assmann B. Clinical Reasoning: Shuddering attacks in infancy. Neurology. 2008;70(13). DOI: 10.1212/01.wnl.0000306698.75592.6e 18362276

[14] Nakamura K, Morrison SF. Central efferent pathways for cold-defensive and febrile shivering. J Physiol. 2011;589(Pt 14):3641–3658. DOI: 10.1113/jphysiol.2011.210047 PMC3167123 21610139

[15] Morrison SF. Central control of body temperature. F1000Research. 2016;5:F1000 Faculty Rev-880. DOI: 10.12688/f1000research.7958.1

[16] Bell DG, Tikuisis P, Jacobs I. Relative intensity of muscular contraction during shivering. J Appl Physiol. 1992;72(6):2336–2342. DOI: 10.1152/jappl.1992.72.6.2336 1629089

[17] Israel DJ, Pozos RS. Synchronized slow-amplitude modulations in the electromyograms of shivering muscles. J Appl Physiol. 1989;66(5):2358–2363. DOI: 10.1152/jappl.1989.66.5.2358 2745301

[18] Pozos RS, Israel D, McCutcheon R, Wittmers LE, Sessler D. Human studies concerning thermal-induced shivering, postoperative “Shivering”, and cold-induced vasodilation. Ann Emerg Med. 1987;16(9):1037–1041. DOI: 10.1016/S0196-0644(87)80756-4 3631668

[19] Tikuisis P, Bell DG, Jacobs I. Shivering onset, metabolic response, and convective heat transfer during cold air exposure. J Appl Physiol. 1991;70(5):1996–2002. DOI: 10.1152/jappl.1991.70.5.1996 1864780

[20] Darocha T, Pasquier M, Mendrala K, et al. Accidental hypothermia. Nat Rev Dis Primer. 2026;12(1):43. DOI: 10.1038/s41572-026-00717-7 42393082

[21] Sund-Levander M, Wahren LK. Assessment and prevention of shivering in patients with severe cerebral injury. A pilot study. J Clin Nurs. 2000;9(1):55–61. DOI: 10.1046/j.1365-2702.2000.00352.x 11022493

[22] Aita T, Nakagawa H, Takahashi S, et al. Utility of shaking chills as a diagnostic sign for bacteremia in adults: a systematic review and meta-analysis. BMC Med. 2024;22(1):240. DOI: 10.1186/s12916-024-03467-z PMC11167933 38863066

[23] The American Board of Pediatrics, Research Department. General Pediatrician Age/Gender Distribution and Summary. https://www.abp.org/content/data-and-workforce. 2026.

